# Platinum Nanoparticle Extraction, Quantification, and Characterization in Sediments by Single-Particle Inductively Coupled Plasma Time-of-Flight Mass Spectrometry

**DOI:** 10.3390/nano12193307

**Published:** 2022-09-23

**Authors:** Sara Taskula, Lucie Stetten, Frank von der Kammer, Thilo Hofmann

**Affiliations:** Department of Environmental Geosciences, Centre for Microbiology and Environmental Systems Science, University of Vienna, 1090 Vienna, Austria

**Keywords:** multi-element single-particle inductively coupled plasma time-of-flight mass spectrometry, nanoparticle extraction, platinum nanoparticles, elemental composition

## Abstract

Particulate emissions from vehicle exhaust catalysts are the primary contributors to platinum group elements (PGEs) being released into roadside environments, especially platinum (Pt) particles. With increasing traffic density, it is essential to quantify the emission, accumulation, and potential health effects of traffic-emitted Pt particles. In this study, three procedures were investigated to extract Pt nanoparticles (NPs) from sediments and characterize them by single-particle inductively coupled plasma time-of-flight mass spectrometry (spICP-TOF-MS). For this purpose, a reference sediment sample was spiked with manufactured Pt NPs. Pt NPs’ extraction recoveries reached from 50% up to 102%, depending on the extraction procedure and whether the particle mass or number was used as the metric. Between 17% and 35% of the Pt NPs were found as unassociated Pt NPs and between 31% and 78% as Pt NPs hetero-aggregated with other sediment particles. Multi-elemental analysis of Pt-containing NPs in the pristine sediment revealed frequently co-occurring elements such as Au, Bi, and Ir, which can be used to determine a natural background baseline. Our results demonstrated that spICP-TOF-MS elemental characterization allows for distinguishing anthropogenic Pt NPs from the natural background. In the future, this could enable the sensitive monitoring of PGE release from anthropogenic sources such as vehicle exhausts.

## 1. Introduction

Traffic emissions are considered to be the main contributors to anthropogenic particulate matter (PM) in urban air and roadside environments, varying from 5% to 61% of the total air pollution depending on the traffic density [1]. Coarse particles (PM_10_: 2.5 µm–10 µm) are derived mainly from non-exhaust sources such as brake and tire wear and road dust resuspension [2]. By contrast, fine (PM_2.5_: 0.1 µm–2.5 µm) and ultra-fine (PM_0.1_: <0.1 µm) particles are predominantly emitted from vehicle exhausts [2]. Traffic-related particle emissions have been linked to adverse health effects [2,3,4,5]. In particular, fine and ultra-fine particles present a substantial risk, as they can enter deep into the respiratory system and the bloodstream [2,3,4,5]. The release of fine and ultra-fine particle emissions has been linked to an increase in cardiovascular and respiratory illnesses such as asthma, reduced lung function, lung cancer, and respiratory infections [5].

Ultra-fine PM_0.1_ particles (nanoparticles (NPs)) have a wide range of compositions, depending on their origins [6]. Commonly released from vehicle exhaust are NPs of platinum group elements (PGEs), including platinum (Pt), palladium (Pd), and rhodium (Rh), because of their use in automotive catalytic converters to reduce the emission of toxic gases [7]. They are predominantly (>95%) released in particulate form, either as single NPs or associated with other exhaust particles containing elements such as Ce, Pb, Zn, and Zr [8,9,10]. Being released close to road surfaces, the majority of particles are deposited in roadside environments, up to 100 m from the road [9,11,12,13]. Road run-off transfers these particles further into surface waters, sediments, and soils or, when a combined sewer system is present, into sewage treatment plant sludge [14,15,16,17,18]. Elevated Pt concentrations have been consequently reported in urban and industrialized environments. For example, Pt concentrations of 1–20 pg·m^−3^ have been found in urban air, of 50–300 ng·g^−1^ in street dust, of 20–250 ng·g^−1^ in roadside soils, of 1–60 ng·g^−1^ in sediments, and of 1–10 ng·L^−1^ in urban water [12,19].

The natural background amount of Pt is low, with an average concentration of 0.6 ng·g^−1^ in the continental crust [20]. In anthropogenically unimpacted soils, Pt concentrations are variable: 47 pristine Italian soils derived from volcanic rock, sandstone, limestone, and other parent materials showed Pt concentrations between 0.1 ng·g^−1^ and 8.4 ng·g^−1^ [11]. Establishing a universal natural background concentration and determining accurately the anthropogenic contribution locally is hampered by this variation and the fact that little data are available from the time prior to the widespread implementation of catalytic converters. The continuing accumulation of PGEs in urban soil and sediments, however, leads to Pt concentrations (20–250 ng·g^−1^; [12,19]) which show undoubtedly an anthropogenic impact even when the precise contribution remains inaccessible. In soils and sediments, Pt forms have been identified as complexes with inorganic and organic ligands or as nano- and microparticles associated with organic and/or mineral phases or accumulated by biota [14].

The small size and low concentration (ng·g^−1^) of Pt-containing particles in natural environments make their detection and characterization challenging [21]. The quantitative extraction of Pt-containing NPs from environmental matrices and sensitive analytical techniques able to quantify particle elemental composition, ideally being able to distinguish between natural and anthropogenic sources, are therefore crucial to elucidate the environmental pollution by PGE NPs from automotive catalysts and their transport, distribution, and transformation.

Inductively coupled plasma quadrupole and sector-field mass spectrometers (ICP-QMS and ICP-SFMS) operated in a time-resolved mode are commonly used to analyze individual inorganic (nano-)particles [21]. To obtain the required time resolution, these mass spectrometers are in general locked on one isotope, which does not permit multi-elemental characterization of individual particles. Fast isotope switching may provide access to two or three isotopes, but at the cost of lower elemental quantification capabilities. In contrast, an ICP-MS equipped with a time-of-flight mass spectrometer (ICP-TOF-MS) can quasi-simultaneously detect elements over the entire mass range (7–275 m/z) in extremely short time intervals of less than 50 µs, permitting multi-elemental analysis of individual NPs [22]. This emerging technique has opened up the possibility of distinguishing engineered nanoparticles (ENPs) from natural nanoparticles (NNPs), as shown by Praetorius et al., who discerned CeO_2_ ENPs and Ce-containing NNPs in soils by single-particle multi-element fingerprinting [23]. Likewise, Bevers et al. used single-particle ICP-TOF-MS (spICP-TOF-MS) to characterize Zn NPs in urban watersheds [24], and Loosli et al. identified elemental associations of TiO_2_ ENPs and NNPs in surface waters and sewage spills [25].

Particle extraction procedures have been shown to be the critical step in sample preparation for particle analysis in soils or sediments. Several physical and chemical extraction procedures have been used to extract and disperse nanomaterials from natural samples for particle analysis [26,27,28,29,30]. A colloidal extraction procedure was developed by Plathe et al. to extract NNPs from river sediments [30]. This procedure has subsequently been adapted and optimized to extract CuO and CeO_2_ NPs from soils [23,27,31], and TiO_2_ NPs from lake sediments [32]. TiO_2_ and CeO_2_ NPs have also been extracted from sediment and soil samples using dispersing agents such as NaOH, Na_2_C_2_O_4_, Na_4_P_2_O_7_, and FL70 detergent to detach and disperse the extracted NPs and to stabilize them against aggregation [28,29].

In this study, three procedures were compared regarding their efficiency in extracting Pt-containing NPs from sediments. A pristine and certified lake sediment was spiked with manufactured Pt NPs, and extraction efficiencies were evaluated. Physical and colloidal extractions were performed. Centrifugation was applied to obtain an upper particle size cut-off, below which particles can be quantitatively ionized by the ICP of the ICP-TOF-MS. Upper particle size limits between 1 and 5 μm are recommended for complete ionization of the particles in the plasma [27,33,34,35]. The laboratory detergent FL70 was used to stabilize the extracted particles and enhance the extraction efficiency. Multi-element analysis of the spiked Pt NPs by spICP-TOF-MS revealed that the Pt NPs were extracted as single/unassociated NPs or as NPs hetero-aggregated with other sediment particles. Additionally, the elemental compositions of Pt-containing NPs extracted from the pristine sediment were investigated to test whether a natural background could be established. We show that an almost complete extraction of Pt NPs from Pt-spiked sediments can be achieved and that spICP-TOF-MS represents a powerful tool for detecting and characterizing Pt-containing NPs in sediments.

## 2. Materials and Methods

### 2.1. Nanoparticle Extraction Procedure

**Figure 1:**
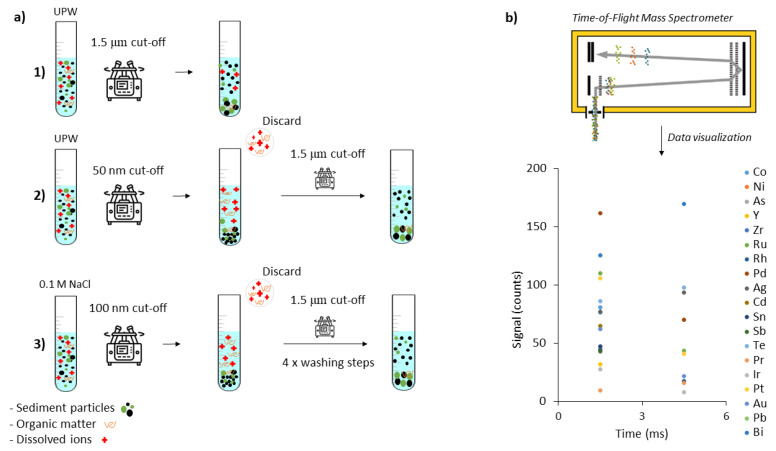
(**a**) Scheme of procedures to extract Pt NPs from the sediment samples: (1) physical one-step, (2) physical two-step, and (3) colloidal extraction procedure. (**b**) Particle detection by spICP-TOF-MS and processed data visualization.

Nanoparticle extraction procedures were performed on the certified reference material LKSD-1 [36], consisting of lake sediments collected from Joe Lake and Brady Lake in Ontario, Canada. Catalytic converters were implemented in cars worldwide in 1970s, and the sediment samples were collected 20 years after, in 1990 [37]. Due to the rural location of the lakes, the LKSD-1 material likely contains little to no traffic-related particles. It is therefore a good model to represent natural sediments, unimpacted by anthropogenic Pt NP inputs and containing no or only a small amount of anthropogenic Pt NPs. Briefly, 50 nm Pt NPs (Table S1) (NanoExact by NanoComposix, San Diego, CA, USA) were dispersed in 10 mL of UPW, resulting in a Pt NPs suspension of 26.5 ng·mL^−1^; 100 µL of the Pt NPs suspension was then added to 250 mg of sediments and vigorously mixed by vortexing (Vortex-GENIE 2, Scientific Industries, Bohemia, NY, USA. The Pt NPs were then extracted according to the extraction procedures described below (Figure 1).

Three procedures to extract Pt-containing NPs from sediment samples were compared (Figure 1). For each sample, the extraction procedures were performed in duplicate (Table S2).

The physical one-step extraction aimed to physically extract particles from the suspended bulk material by ultrapure water and sonication. Briefly, 250 mg of milled dry sediment was mixed with 10 mL of ultrapure water (UPW: Milli-Q, ELGA PURELAB Chorus, High Wycombe, UK), vortexed, sonicated for 3 min at 30 W (VialTweeter ultrasonic processor, Hielscher UP200ST, Teltow, Germany), and centrifuged at 1000× *g* for 6 min (Sorvall LYNX 6000, Thermo Scientific, Waltham, MA, USA. According to Stokes’ law, the centrifugation step led to a 1.5 µm particle size cut-off, assuming particles with a density of 2.6 g·cm^−3^ and a spherical shape [30]. In the case of pure Pt NPs with a density of 21.45 g·cm^−3^ [38], this centrifugation step led to a 0.43 µm particle size cut-off. The supernatant was carefully decanted and diluted by a factor of 200 in either UPW or 0.005% FL70 (SF105-1, Fischer Scientific, Waltham, MA, USA) to investigate the stability of the extracted NPs. The latter dispersions were bath sonicated for 3 min (Bandelin Sonorex Super RK 106, Berlin, Germany) prior to single-particle analysis.

The physical two-step extraction employed the same scheme as the one-step extraction but added a washing step to decrease the concentration of dissolved background species in the final particle extract. Dissolved species create a high load on the detector, which requires a dilution of the sample, which in turn reduces the number concentration of the target particles in the extract. Another unwanted side effect of a high amount of dissolved ionic background material is the aggregation of the particles in the extract. For this purpose, 250 mg of dry milled sediment was mixed with 10 mL of UPW. The mixture was shaken, sonicated (30 W, 3 min), and centrifuged at 5500× *g* for 1 h. This centrifugation step led to a 50 nm particle size cut-off, assuming particles with a 2.6 g·cm^−3^ density, and a 20 nm particle size cut-off for pure Pt particles. The supernatant was discarded, and the sediment was resuspended in 10 mL UPW, vortexed, sonicated, and centrifuged to a 1.5 µm particle size cut-off (1000× *g* for 6 min). The supernatant was then collected, diluted by a factor of 200 in either UPW or 0.005% FL70, and bath sonicated for 3 min prior to single-particle analysis.

The colloidal extraction procedure was adapted from Plathe et al. [30]. It aimed to increase the sodium adsorption ratio (i.e., the relative activity of sodium ions to calcium and magnesium ions) to improve particle dispersion and remove divalent cations such as Ca^2+^ that affect the stability of NPs and induce aggregation [30]. About 250 mg of dry milled sediment was mixed with 10 mL of 0.1 mol·L^−1^ NaCl, vortexed, sonicated for 3 min at 30 W, and centrifuged to a 100 nm particle size cut-off (5500× *g* for 46 min, assuming a density of 2.6 g·cm^−3^ for the particles). In the case of pure Pt particles, the particle size cut-off was 28 nm. The supernatant was discarded, and the sediment was washed four times in total by resuspending it in 10 mL UPW, vortexing, sonicating (30 W, 3 min), and centrifuging as before. The washed sediment was then resuspended, vortexed, sonicated, and centrifuged to a 1.5 µm particle size cut-off (1000× *g* for 6 min). The final extract was diluted by a factor of 200 either in UPW or in 0.005% FL70 and bath sonicated for 3 min prior to single-particle analysis.

### 2.2. Pt Nanoparticle Extraction Efficiency

The Pt-spiked sediment samples were used to evaluate the efficiency of each extraction procedure. The extraction efficiency was estimated after background subtraction by two different methods, one based on the number of particles recovered (the particle number recovery: NP_%_) and one based on the mass of particles recovered (the mass recovery: M_%_). The background was evaluated by measuring the average number concentration (C_Background_) and mass (M_Background_) of Pt-containing NPs in the pristine sediment samples.

The particle number recovery method (NP_%_) used the ratio of the particle number concentration of Pt NPs detected (C_NP_detected_) to the measured particle number concentration of Pt NPs spiked in the sample (C_NP_spiked_ = 820 ± 150 NP·mL^−1^):

NP_%_ (%) = (C_NP_detected_ − C_Background_)/C_NP_spiked_ × 100. (1)

C_NP_spiked_ was calculated from the particle number concentration measured by spICP-TOF-MS (C_NP_measured_ = 3.29 × 10^10^ ± 5.99 × 10^9^ NP·mL^−1^, Table S1), the volume of the spiked solution (V_spiked_ = 100 μL), and the dilution factors (DF) as C_NP_spiked_ = C_NP_measured_ × V_spiked_ × DF.

The mass recovery method (M_%_) used the ratio of the total mass of Pt NPs detected (M_detected_) to the measured total mass of spiked Pt NPs in the sample (M_spiked_ = 0.65 × 10^−3^ ± 0.12 × 10^−3^ ng·mL^−1^):

M_%_ (%) = (M_detected_ − M_Background_)/M_spiked_ × 100. (2)

M_spiked_ was calculated from the mass concentration measured by spICP-TOF-MS (M_measured_ = 26.1 × 10^3^ ± 4.74 × 10^3^ ng·mL^−1^, Table S1), the volume of the spiked solution (V_spiked_ = 100 μL), and the dilution factors (DF) as M_spiked_ = M_measured_ × V_spiked_ × DF.

### 2.3. Single-Particle ICP-TOF-MS Analysis

Single-particle analyses were performed using an ICP-TOF-MS instrument (icpTOF 2R, TOFWERK AG, Thun, Switzerland). A detailed description of the instrument can be found elsewhere [22,39,40]. Operational parameters can be found in Table S3. In this study, the samples were introduced through a desolvation membrane system (Table S4), consisting of a concentric pneumatic nebulizer combined with a membrane desolvation unit (Apex Omega, ESI, Omaha, NE, USA). By producing a dry aerosol, solvent-generated interferences, such as oxide and hydroxide interferences, were reduced, and the signal intensity was maximized. Each Pt-spiked sediment dispersion was measured for 1 min, and the pristine sediment dispersions for 1, 2, and 5 min (Table S2).

For instrument calibration, dissolved calibration standards were prepared from single-element solutions (Inorganic Ventures, Christiansburg, VA, USA). Elements with no known interferences were grouped into multi-element standard batches. The first batch [Au, Ir, Pd, Pt, Rh, Ru, Te] was prepared in 1 wt% HCl (ROTIPURAN Ultra, ROTH, Karlsruhe, Germany). The other three batches [Ba, Cd, Ce, Co, Cr, Pr, V, Y, Zr], [As, Bi, Cu, La, Mo, Ni, Pb, Sb, Sn, Zn], and [Al, Fe, Mg, Mn, Na, Si, Ti], were prepared in 2 wt% HNO_3_ (ROTIPURAN Ultra, ROTH, Karlsruhe, Germany). The transport efficiency was determined with the particle size method as in Pace et al. [41], using 100 nm Au NPs (BBI Solutions, Crumlin, UK) in UPW and dissolved Au standards in 1 wt% HCl. The autotuning module of the control software TOFpilot (V2.8, TOFWERK AG, Thun, Switzerland) was used to tune and optimize the instrument for the best sensitivity and resolution as well as the lowest oxide (CeO^+^/Ce^+^) and doubly charged (Ba^++^/Ba^+^) levels (Table S3) using a solution of 1 μg·L^−1^ Ba, Bi, Ce, Co, In, Li, and U (Thermo iCAP Q, Thermo Scientific, Waltham, MA, USA). The sensitivity and resolution of ^59^Co, ^115^In, and ^238^U were monitored before, during, and after each measurement to account for any drift in instrument sensitivity. The sensitivities and resolutions were stable throughout the measurement time and within the average values commonly achieved with the ICP-TOF-MS (Figure S1).

Single-particle analyses were performed with the single-particle workflow featured in the TOFpilot software, which provided experimental set-up and data processing and quantification. The software determined mass calibration curves using liquid standards, transport efficiency using the particle size method, limits of detection, particle number concentrations, and mass distributions all according to Pace et al. [41]. The collected data were processed after the measurements using the TOFpilot liquid reprocessing module (TOFWERK AG, Thun, Switzerland). For every single isotope, particle signals were separated from the background by performing iterative signal/background separation. A window of 100 data points was used, and the threshold was determined according to Equation (3) [42]

(3) Threshold= Avg+(3.29×SD)+2.71.

The average (Avg) and standard deviation (SD) were calculated for each window. Signals above the threshold were selected and extracted as particle signals, and the iterations continued until no more peaks were detected. The ICP-TOF-MS operational parameters for each measurement can be found in Table S3. The limits of detection (LOD) of all monitored isotopes were determined using acidified calibration standards with the 3 sigma formula [43] (Table S5). The LOD of Pt was 4.55 ng·L^−1^, corresponding to 0.014 fg and 11 nm, according to Pace et al. [41]. Considering the dilution factors, the LOD corresponded to a method limit of detection of 1.77 × 10^−3^ ng·g^−1^ for Pt. The particle number concentration limit of detection (LOD_NP_) was determined as LOD_NP_ = 3/(Transport efficiency × Flow rate × Total acquisition time) [33,44] and corresponded to 4.88 × 10^4^ L^−1^. Current ICP-MS instruments have LOD_NP_ values in the range of 10^6^ L^−1^ [33,44,45].

### 2.4. Mineral and Carbon Analysis

The mineralogy of the sediment LKSD-1 was determined qualitatively by powder X-ray diffraction using a Rigaku Miniflex 600 diffractometer (Rigaku, Tokyo, Japan) equipped with a monochromatized source of Cu Kα radiation (λ = 0.15405 nm) (Figure S2). A step size of 0.03° over the 2θ scan range 5–85° and a scanning rate of 2°·min^−1^ were used to record the X-ray diffraction patterns, and the minerals were identified using the SmartLab Studio II software (Ver.1.4.3, Rigaku, Tokyo, Japan).

Total organic carbon (TOC) and inorganic carbon (TIC) contents in the sediment sample were determined with a LECO-TOC RC612 carbon analyzer (LECO, St. Joseph, MO, USA) (Table S6). The TOC content was determined as released carbon dioxide at a temperature ranging from 105 °C to 550 °C, while the TIC content was determined by increasing the temperature from 550 °C to 1000 °C. Before the sample analysis, the background carbon within the system was determined, followed by instrument calibration with known calibration standards (CaCO_3_ and synthetic carbon).

## 3. Results and Discussion

### 3.1. The Pristine Sediments and Natural Pt-Containing NPs

In the pristine LKSD-1 sediment, only a few Pt-containing NPs were detected in the particle extracts (Table S2). Between 1 and 4 Pt-containing NPs were identified within a total of 5387–5697 particles, compared to between 40 and 72 in the Pt-spiked sediment (1 min measurement each, Table S2). This particulate Pt background corresponded to 2% to 5% of the Pt NPs detected in the Pt-spiked samples. Those particles were unexpected and are likely not related to car exhaust but represent the natural Pt-containing particle background, as we will show later. This natural background was considered in the Pt NP recovery calculations. One Pt NP was detected in one FL70 blank, and none were detected in one UPW blank (Table S2), indicating that particle carry-over can occur, but only rarely.

Using the multi-elemental characterization capabilities of the spICP-TOF-MS, the elemental compositions of Pt-containing NPs in the unspiked/pristine sediment were determined to investigate common elemental compositions of Pt-containing NPs believed to have natural origins. The physical one-step extraction procedure was applied to the sediment samples, using FL70 as the dispersant, as it proved to be suitable for extracting Pt-containing NPs from Pt-spiked sediments (as we will show later, Section 3.2). The number of natural Pt-containing NPs was very low in the pristine sediment and sediment extracts. This required extending the measurement time to 10 min in total to capture a sufficient number of Pt-containing particles representing the natural background in the sediment. A total of 90 Pt-containing NPs were detected, which was estimated to an average total mass concentration of 0.50 ± 0.37 ng·g_sediment_^−1^, assuming 100% NP recovery. This value is within the range of Pt concentration in the continental crust (0.6 ng·g^−1^; [20]) and natural soils and sediments (0.1–8.4 ng·g^−1^; [11,14,46]).

**Figure 2:**
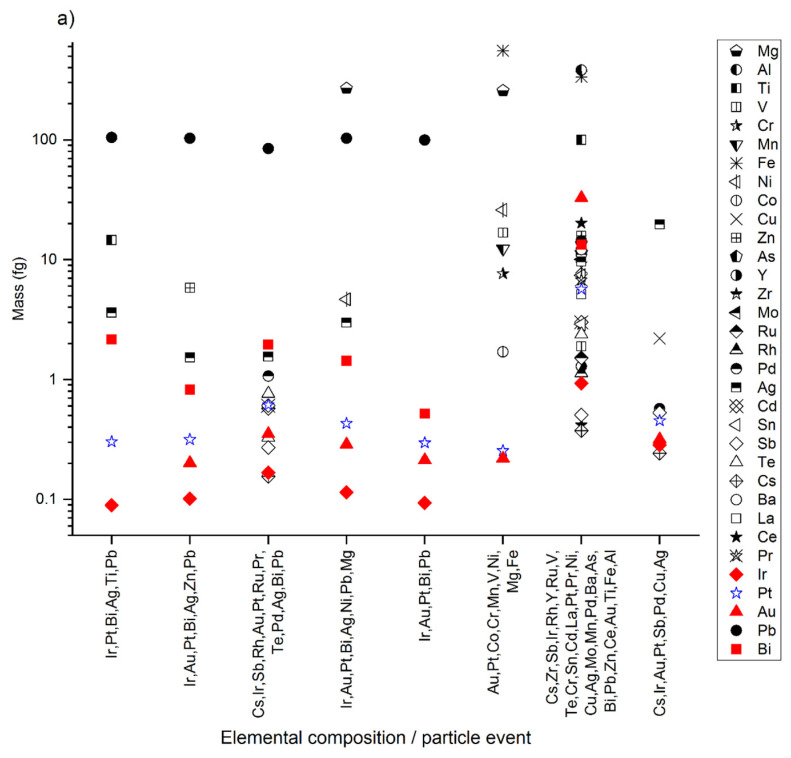

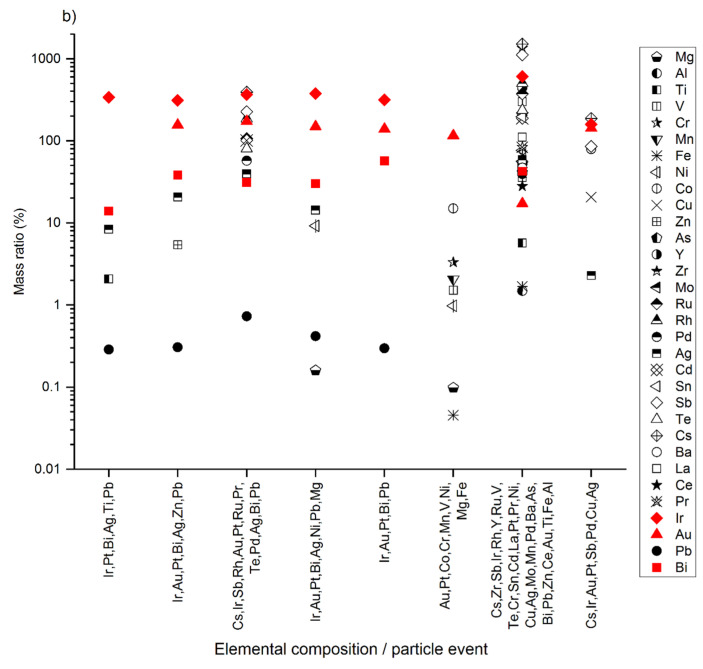
(**a**) Pt-containing NP elemental compositions and (**b**) mass ratio of Pt to other elements in the Pt-containing NPs in the unspiked/pristine sediment sample. The NPs were extracted by physical one-step extraction, dispersed in FL70, and acquired over a period of 2 min out of 10 min. The *x*-axis indicates the elemental composition of each particle represented. Pt is marked in blue and Ir, Au, and Bi in red to facilitate their perception.

For all detected Pt-containing NPs, between 5 and 31 chemical elements were detected in association with Pt (Figure 2). Ir, Au, and Bi were detected in most of the Pt-containing NPs in the pristine sediment (Figure 2). Furthermore, 92% of the detected NPs contained Ir, 75% contained Au, and 81% contained Bi in addition to Pt (Table 1); 72% of the NPs contained Ir and Au, and 66% contained Ir, Au, and Bi together (Table 1). Furthermore, the average mass ratios of Pt/Ir and Pt/Au were within the same range for all detected Pt-containing NPs (Pt/Ir = 3.38 ± 0.92 and Pt/Au = 1.54 ± 0.21; Table 1). By contrast, the mass ratio of Pt/Bi varied broadly among all detected Pt-containing NPs (Pt/Bi = 0.96 ± 1.40; Table 1). Praetorius et al. showed the potential of using specific chemical elements and ratios such as rare earth elements to identify Ce-containing NNPs [23]. Ce-containing NNPs were identified by the presence of rare earth elements (La, Pr, Nd, and Ba), with a Ce/La ratio of 2, whereas engineered Ce NPs contained Ce only. The presence of Ir, Au, and Bi in engineered Pt-containing NPs has, to date, not been reported. Our results suggest that Ir, Au, Bi, and/or the latter’s elemental ratios can be used as indicators for natural Pt-containing NPs. However, more data in terms of particle number and sample diversity are required to identify elemental fingerprints.

For all Pt-containing NPs detected, the masses of Pt and other PGEs (i.e., Ir, Pd, Rh, and Ru) were much smaller (<1 fg; Figure 2a) than those of, for example, Al, Mg, Ni, Pb, Ti, Zn, and V (10–100 fg; Figure 2a). Consistently, the mass ratios of Pt to PGEs were high (>0.3; Figure 2b), whereas for Pt to Al, Mg, Ni, Pb, Ti, Zn, and V, the ratios were low (<0.1; Figure 2b). In soils and sediments, Pt is generally found as Pt complexes and Pt NPs associated with mineral phases and organic matter [14,47]. The trace content of Pt and other PGEs in the detected particles might indicate that the particles contain Pt in the form of sorbed species and/or as very small Pt NPs associated with much larger particles and/or in trace amounts in host minerals.

### 3.2. Extraction of Spiked Pure Pt NPs

**Figure 3:**
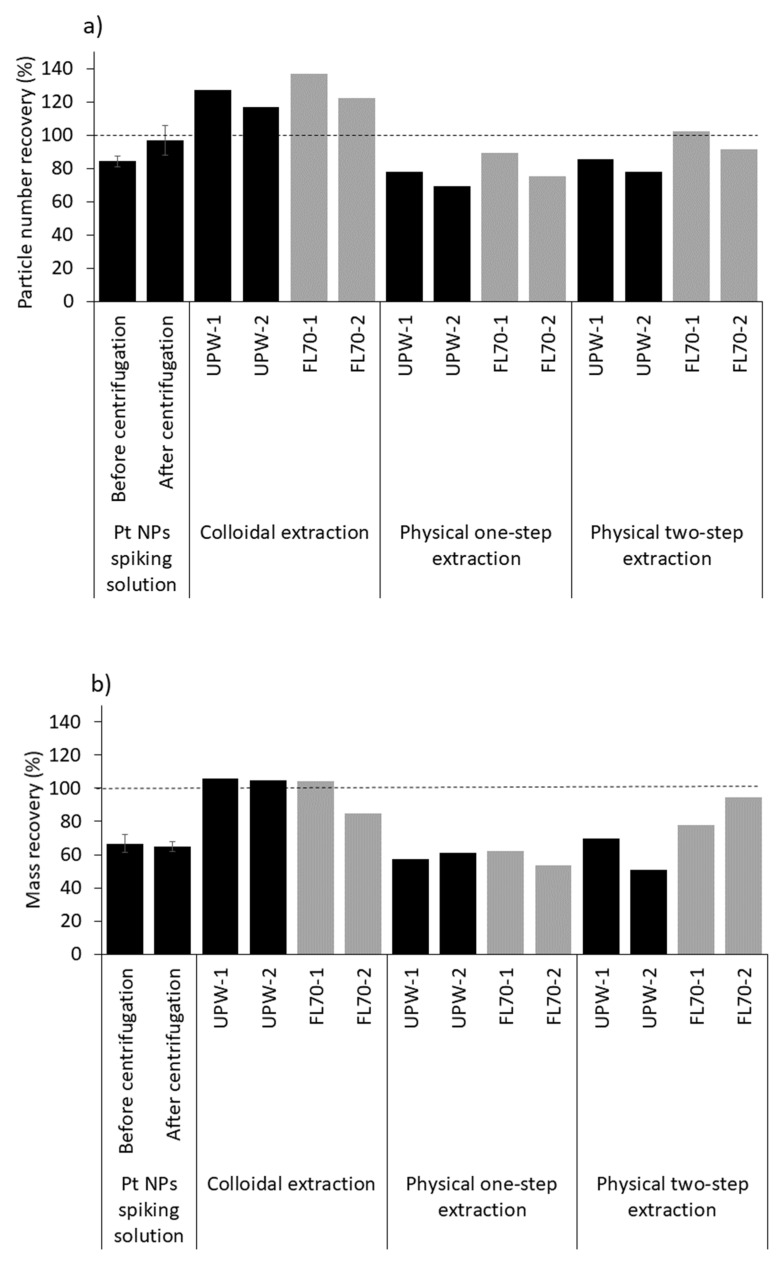
(**a**) Pt particle number recoveries (NP_%_) and (**b**) Pt mass recoveries (M_%_) calculated in the Pt-spiked sediment samples for each extraction procedure using UPW or FL70 as a stabilizer, and in the Pt NP spiking solutions before and after centrifugation to a 1.5 μm cut-off, dispersed in UPW (Table S2). Each sample was prepared and measured independently for 1 min. The particle concentration given by the manufacturer (4.48 × 10^10^ NP·mL^−1^, Table S1) was higher than the mean particle concentration measured in the spiking solution (3.29 × 10^10^ NP·mL^−1^, Table S1), leading to the low recovery value from the spiking solution before and after centrifugation.

The extraction efficiencies are given in Figure 3 and Table S2. For the colloidal extraction procedure, particle number recoveries between 117% and 137% were obtained from the particle extracts in FL70 and UPW duplicates (Figure 3a). This method was previously shown to be suitable for extracting CuO NPs from soil and sediment samples [27,30]. The physical one-step and two-step extraction procedures yielded slightly lower recoveries ranging between 59% and 90% and between 78% and 102%, respectively (Figure 3a). Although the physical one-step extraction procedure had the lowest recovery, it may be suitable for screening purposes or when dealing with a large number of samples because it is an easy and fast procedure.

The variabilities between the initial measurements and the measurements reported in this manuscript for the one-step extractions were 6% and 9% in UPW and FL70, respectively. For the physical two-step extractions, they were 5% and 8% in UPW and FL70, respectively. The variabilities between the physical one-step and two-step extractions were 0.6% and 1% for UPW and FL70, respectively. Therefore, the difference between the physical one-step and two-step extractions is not significant.

For all extraction procedures, the mass recoveries were lower than the particle number recoveries (Figure 3b). The average mass of recovered Pt NPs from the Pt-spiked sediments was 0.60 ± 0.42 fg (Figures S5–S8) and within the range measured in the Pt NP spiking solution (0.90 ± 0.46 fg; Figures S3 and S8). Additionally, the mass distribution of Pt in the Pt NP spiking solution was evaluated after centrifuging the solution to a 1.5 μm cut-off. It shows a different pattern, with a mass distribution of Pt particles shifted to smaller masses (M_Pt-average-centrifuged_ = 0.78 ± 0.32 fg; Figure S4) compared to the Pt mass distribution determined before centrifugation (M_Pt-average_ = 0.90 ± 0.46 fg; Figure S3). Considering these results, the lower mass recoveries compared to the particle number recoveries could be interpreted as the loss of large, high mass Pt NPs (>1.7 fg) following centrifugation (to the 1.5 μm cut-off) and resuspension, which shifts the average particle mass to a lower value. The detected and analyzed Pt-containing NPs would therefore represent the small mass/size fraction of the spiked Pt NPs.

The same applies to “real-world” NPs, and the smaller mass/size fraction may be predominantly extracted and detected using the studied extraction procedures. Using a higher particle size cut-off at the centrifugation step may reduce particle size fractionation and extract larger Pt-containing NPs.

Recoveries were slightly higher when using FL70 compared to UPW as a dispersant for all procedures (Figure 3). The FL70 concentration used in this study was low (0.005%) to avoid overloading the detector of the ICP-TOF-MS. This value is much lower than the concentrations previously used to disperse NPs extracted from soils and sediments, ranging between 0.01% and 0.1% [48,49,50], showing that even low concentrations of FL70 can improve the stabilization of extracted Pt NPs in the particle extracts compared to UPW alone.

An elevated aqueous ionic background was observed in the particle extracts compared to UPW and FL70 blanks; in particular, the concentrations of aluminum, calcium, iron, and magnesium (Table S7) were increased. The centrifugation and resuspension step and the removal of the supernatant in the physical two-step extraction procedure aimed to reduce the dissolved concentrations in the final particle extract in order to decrease the detector load and therefore prevent high dilution factors for the particle extracts prior to analysis. Less dilution of the particle extracts would increase the number of particles detected per measurement cycle of 1 min and is more appropriate for sediments containing low concentrations of Pt-containing NPs. In the colloidal extraction procedure, the several centrifugation and resuspension steps aimed to lower the high ionic strength induced by the addition of NaCl and thus reach an ionic strength where most of the colloidal soil particles would go into dispersion. However, no reduction in the total dissolved ion concentrations of the supernatants was observed. No definite reason was found for our observations. Dissolution of calcite (Figure S2) can lead to a high Ca^2+^ ion background. Ionic exchange between organic matter (TOC = 10.7%; Table S6) and metal ions or minerals [51] can also influence the concentrations of dissolved ions. However, we did not observe any change in the concentrations of Ca^2+^ or other ions in the particle extracts, with the pH ranging between 7 and 9.

The centrifugation and resuspension steps of the extraction procedures have two different purposes. The 1.5 µm centrifugation step aims at the supernatant and the removal of oversized particles which cannot be correctly analyzed by the ICP-TOF-MS. The 50–100 nm step aims to remove all particles from the sediment; here there is a small risk of losing some very small particles in the discarded supernatant, but the procedure will recover at least all larger particles and all pure Pt particles larger than 28 nm due to their higher density. This might lead to losses of Pt NPs and the incomplete extraction of the spiked Pt NPs. Moreover, some Pt NPs may fall below the size detection limit of the ICP-TOF-MS (0.014 fg corresponding to 11 nm).

### 3.3. Extracted Pure Pt NPs Appear as Unassociated and Hetero-Aggregated NPs

**Figure 4:**
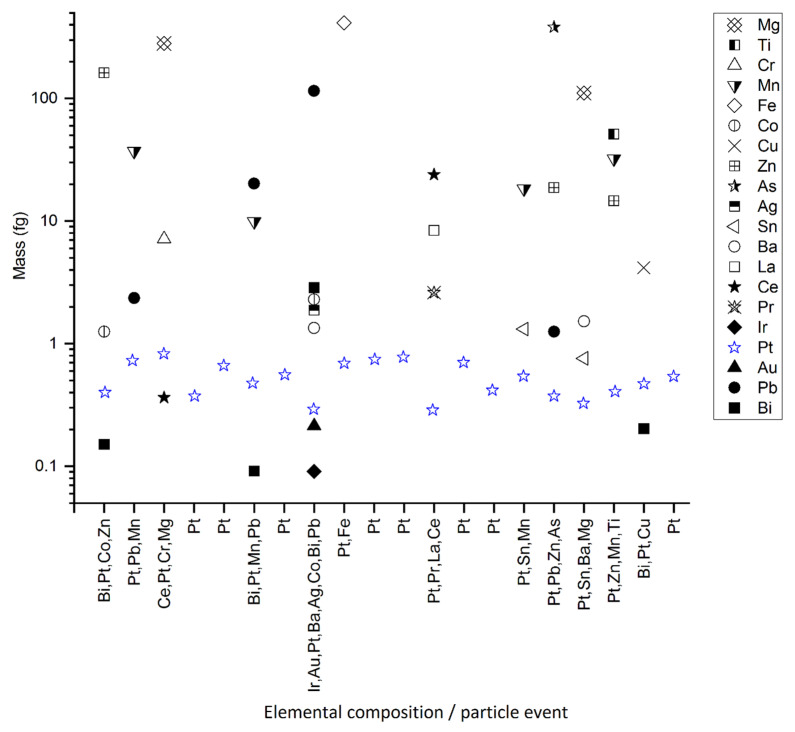
Elemental compositions of the detected Pt NPs in the Pt-spiked sediment dispersion. The graph shows the NPs extracted by colloidal extraction, dispersed in FL70, and acquired over a period of 20 s out of 1 min total acquisition time. The *y*-axis indicates the mass of each element and the *x*-axis the elemental composition of the particle.

The elemental composition of the extracted Pt-containing NPs was determined in the samples spiked with manufactured Pt NPs. In all measured samples, two types of Pt-containing NP were distinguished: pure Pt NPs, which are free, unassociated spiked particles, and Pt NPs appearing together with various other elements, such as Fe, Mg, Mn, Pb, and Zn (Figure 4 and Figures S9–S13). For all detected Pt-containing NPs, the mass of Pt ranged between 0.3 fg and 1.0 fg, which corresponds to the mass distribution of Pt in the NPs of the spiking solution after the centrifugation (1.5 μm cut-off) and resuspension step (Figures S4 and S8). The detected Pt-containing NPs likely represent the spiked Pt NPs because of the initial low Pt content of the pristine sediment (Section 3.1). The pure Pt NPs are therefore identified as single/unassociated manufactured Pt NPs. By contrast, the various chemical compositions of most of the detected Pt-containing NPs (Figure 4 and Figures S9–S13) suggested that the spiked Pt NPs aggregated with sediment particles. This interpretation is consistent with several studies showing that Pt NPs can aggregate with mineral phases such as kaolinite [52], Fe/Mn-oxides [47,53], and organic matter [14,47,52,53,54]. For example, Reith and Cornelis showed that Pt NPs are stable in soil solutions and that clays, iron oxides, and organic matter represent the main sorbents for Pt NPs in soil solutions [47].

**Figure 5:**
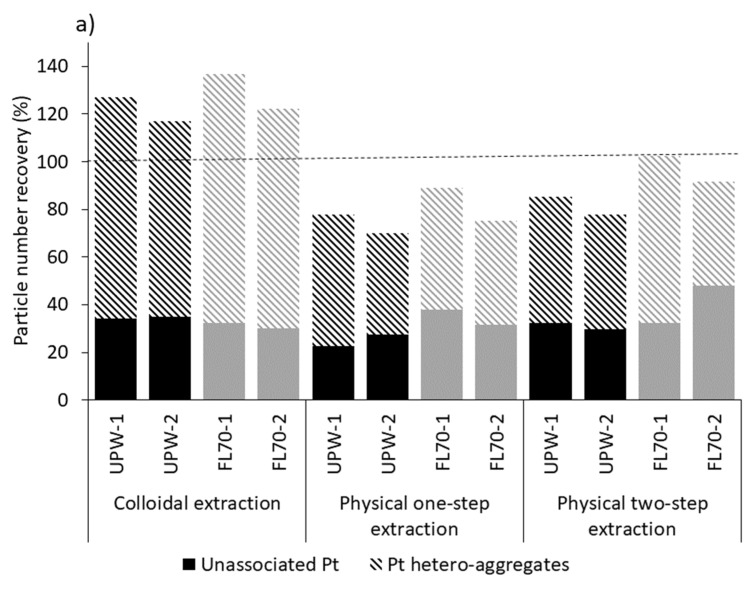

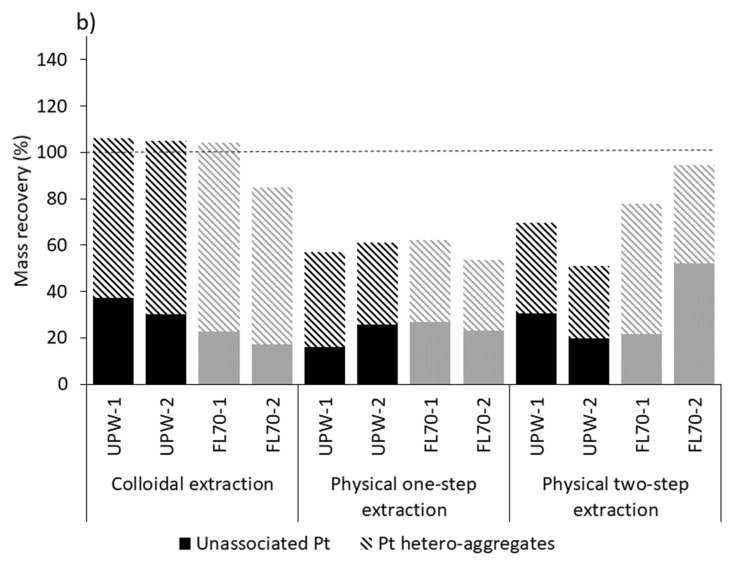
(**a**) Particle number recoveries (NP_%_) and (**b**) particle mass recoveries (M_%_) of unassociated Pt NPs and Pt NPs aggregated with other particles calculated in the Pt-spiked sediment samples for each extraction procedure using UPW or FL70 as a stabilizer.

The extraction efficiencies were evaluated separately for the unassociated Pt NPs and the Pt NPs hetero-aggregated with sediment particles (Figure 5). The particle number recoveries for the various extraction procedures ranged between 23% and 48% for unassociated Pt NPs and between 42% and 104% for hetero-aggregated Pt NPs (Figure 5a). The mass recoveries were lower for both (as observed for the total recoveries; Section 3.2) and showed a similar trend as the particle number recoveries, with a higher recovery of hetero-aggregated Pt NPs (between 32% and 81%) than unassociated Pt NPs (between 16% and 52%) (Figure 5b). These results indicate that the spiked Pt NPs were mainly recovered as hetero-aggregated Pt NPs and suggest that Pt NPs rapidly hetero-aggregate with other particles and form relatively stable aggregates which are not dissociated by sonication.

The recoveries of unassociated Pt NPs were similar for all extraction procedures. By contrast, the colloidal extraction procedure recovered between 82% and 104% of hetero-aggregated Pt NPs, while the physical one-step and physical two-step extractions procedures recovered only between 42% and 70% of hetero-aggregated Pt NPs (Figure 5a). The colloidal extraction especially targets the release and dispersion of clay particles [27,30], such as chlorite (Figure S2), resulting in the extraction of a larger number of sediment particles.

The extracts were stable as dispersions for several hours. However, the agglomeration state of the Pt-containing NPs in the supernatants changed over time. Especially for the Pt-spiked sediment sample Phys1-LKSD-FL70-1, we observed an 18% increase in hetero-aggregated Pt NPs and a 14% decrease in the recovery of unassociated Pt NPs within 5 min of sequential sampling from the same vial (Figure S14). This demonstrates that multi-element single-particle analysis may be used to investigate the hetero-aggregation kinetics of NPs in natural environments [55,56,57,58,59,60]. It would also be possible to determine directly the hetero-aggregation attachment efficiencies of engineered NPs with NNPs, as demonstrated by e.g., Geitner et al., Praetorius et al., and Zhang et al. [56,58,60].

## 4. Conclusions

The development of experimental and analytical methods able to detect, quantify, and characterize Pt-containing NPs at low concentrations, and the investigation of the sources of Pt-containing NPs in natural environments are prerequisites for the monitoring of Pt NP emissions. The complete extraction of NPs from complex matrices such as soils and sediments is particularly important to quantify NPs’ release and potential accumulation, as well as risk assessment. A colloidal extraction procedure with FL70 as a dispersant proved to be best for extracting Pt-containing NPs from sediment samples, with total particle number recoveries of up to 140%. Although the physical one-step extraction resulted in lower total particle number recoveries (up to 90%), it is relatively simple and quick and might be well-suited for screening Pt-containing NPs in sediment samples, especially when high concentrations of the studied NPs are expected. In addition, this latter procedure may limit artifacts resulting from centrifugation steps that potentially lead to size/mass fractionation of the NPs of interest. Multi-elemental single-particle characterization of Pt-spiked sediments by spICP-TOF-MS revealed that Pt NPs readily hetero-aggregate with sediment particles. Pt-containing NPs can be differentiated with regard to their origins based on their individual elemental compositions. This can be proven by combining the results obtained for the pristine sediment samples with those for the Pt-spiked sediment samples.

SpICP-TOF-MS is an efficient tool for determining the elemental ultra-trace compositions of Pt-containing NPs in sediments. This information could be used in future studies to identify and determine elemental fingerprints for natural, anthropogenic, and engineered Pt NPs in the environment. It is a promising approach that can be applied to other PGEs in complex environments such as soils and road dust.

## Figures and Tables

**Table 1:** Proportions of Pt-containing NPs comprising Ir, Au, and Bi from the total 90 Pt-containing NPs detected and the average elemental ratios within the particles in the pristine sediment sample and the Pt-spiked sediment sample (Phys1-LKSD-Spiked-FL70-1). The NPs were extracted by physical one-step extraction, dispersed in FL70, and acquired over a period of 10 min.

Elemental Association		Pt + Ir	Pt + Au	Pt + Bi	Pt + Ir + Au		Pt + Ir + Au + Bi		
**Percent (%)**	**Pristine sediment**	92 ± 4	75 ± 2	81 ± 10	72 ± 2		66 ± 6		
	**Spiked sediment**	6.4	6.4	8.5	6.4		6.4		
		**Pt/Ir**	**Pt/Au**	**Pt/Bi**	**Pt/Ir**	**Pt/Au**	**Pt/Ir**	**Pt/Au**	**Pt/Bi**
**Ratio**	**Pristine sediment**	3.38 ± 0.92	1.54 ± 0.21	0.91 ± 0.48	3.51 ± 0.78	1.54 ± 0.66	3.54 ± 0.78	1.56 ± 0.69	0.96 ± 1.40
	**Spiked sediment**	4.24 ± 0.31	1.70 ± 0.13	1.77 ± 1.77	4.24 ± 0.31	1.70 ± 0.13	4.24 ± 0.31	1.70 ± 0.13	0.89 ± 0.34

## Data Availability

The data presented in this study are available on request from the corresponding author.

## Supplementary Materials

The following supporting information can be downloaded at: https://www.mdpi.com/article/10.3390/nano12193307/s1, Table S1: Manufacturer data and measured values for the 50 nm Pt NPs (NanoComposix, USA); Table S2: Summary of the samples measured in this study; Table S3: ICP-TOF-MS operational parameters on the measurement day of each extraction procedure; Table S4: Apex Omega desolvation unit operation parameters; Table S5: Limits of detection of the monitored analytes converted from concentration LOD_concentration_ (ng·L^−1^) into mass LOD_mass_ (fg) and particle diameter LOD_diameter_ (nm); Table S6: Total organic carbon (TOC), inorganic carbon (TIC), and total carbon (TC) content in the sediment sample; Table S7: Dissolved ion concentration represented as counts per second (cps) of selected major elements in the pristine sediment sample following particle extraction for each extraction procedure; Figure S1: Sensitivity expressed as counts per second (cps); Figure S2: X-ray diffraction pattern of the sediment LKSD-1 sample. Chl: chlorite; Ka: kaolinite; Q: quartz; A: albite; C: calcite; Figure S3: Pt mass distribution of Pt NPs in the undiluted spiking solution; Figure S4: Pt mass distribution of Pt NPs in the spiking solution after centrifugation to a 1.5 μm cut-off, resuspension, and dilution by a factor of 200 in UPW; Figure S5: Pt mass distribution in the Pt NPs of the Pt-spiked sediment sample dispersion. The NPs were extracted by colloidal extraction, dispersed in FL70, and acquired over a period of 1 min; Figure S6: Pt mass distribution in the Pt NPs of the Pt-spiked sediment sample dispersion. The NPs were extracted by physical one-step extraction, dispersed in FL70, and acquired over a period of 1 min; Figure S7: Pt mass distribution in the Pt NPs of the Pt-spiked sediment sample dispersion. The NPs were extracted by physical two-step extraction, dispersed in FL70, and acquired over a period of 1 min; Figure S8: Summary of the mass distribution of Pt in the Pt NPs of the Pt-spiked sediment samples of each extraction procedure and the Pt NP spiking solution before and after centrifugation; Figure S9: Pt-containing NP elemental compositions in the Pt-spiked sediment sample dispersion. The NPs were extracted by physical one-step extraction, dispersed in UPW, and acquired over a period of 30 s out of 1 min total acquisition time; Figure S10: Pt-containing NP elemental compositions in the Pt-spiked sediment sample dispersion. The NPs were extracted by physical one-step extraction, dispersed in FL70, and acquired over a period of 30 s out of 1 min total acquisition time; Figure S11: Pt-containing NP elemental compositions in the Pt-spiked sediment sample dispersion. The NPs were extracted by physical two-step extraction, dispersed in UPW, and acquired over a period of 30 s out of 1 min total acquisition time; Figure S12: Pt-containing NP elemental compositions in the Pt-spiked sediment sample dispersion. The NPs were extracted by physical two-step extraction, dispersed in FL70, and acquired over a period of 30 s out of 1 min total acquisition time; Figure S13: Pt-containing NP elemental compositions in the Pt-spiked sediment sample dispersion. The NPs were extracted by colloidal extraction, dispersed in UPW, and acquired over a period of 30 s out of 1 min total acquisition time; Figure S14: Particle number recoveries (#_%_) of spiked Pt NPs from sediment samples, as single unassociated Pt NPs (black columns) and as Pt NPs hetero-aggregated with other sediment particles (dashed columns), at t = 0 min and t = 5 min after sample redispersion (shaking and sonication).
