# Supplementary material for: Platinum Nanoparticle Extraction, Quantification, and Characterization in Sediments by Single-Particle Inductively Coupled Plasma Time-of-Flight Mass Spectrometry
Source: Nanomaterials (Basel). 2022 Sep 23;12(19):3307. doi: 10.3390/nano12193307 (PMC9565847; doi:10.3390/nano12193307)
Supplement: Supplementary file 1 [file nanomaterials-12-03307-s001.zip › nanomaterials-1835904-supplementary.pdf]

nanomaterials

MDPI

Supplementary Materials

Platinum Nanoparticle Extraction, Quantification, and Characterization in Sediments by Single-Particle Inductively Coupled Plasma Time-of-Flight Mass Spectrometry

Sara Taskula, Lucie Stetten, Frank von der Kammer * and Thilo Hofmann *

Department of Environmental Geosciences, Centre for Microbiology and Environmental Systems Science, University of Vienna, 1090 Vienna, Austria

* Correspondence: frank.von.der.kammer@univie.ac.at (F.v.d.K.); thilo.hofmann@univie.ac.at (T.H.)

Table S1. Manufacturer data and measured values for the 50 nm Pt NPs (NanoComposix, USA).

		Manufacturer	Measured *
**Diameter (TEM)**	nm	46	40
	stdv ±	5	6
**Mass concentration M**	$ng \cdot ml^{-1}$	53.0×10^3	$26.1 \times 10^3 \pm 4.74 \times 10^3$
**Particle concentration C_{NP}**	$NP \cdot ml^{-1}$	4.48×10^{10}	$3.29 \times 10^{10} \pm 5.99 \times 10^9$
**Particle mass M/C_{NP}**	fg	1.18	$7.92 \times 10^{-1} \pm 2.39 \times 10^{-4}$

*Mean value measured by spICP-TOF-MS.

Table S2. Summary of the samples measured in this study.

Particle extraction procedure		Sample	Total number of particles detected	Number of detected Pt NPs	Acquisition time	C_{NP} (#·ml^{-1})
**Physical one-step extraction**	LKSD-1	Phys1-LKSD-UPW-1	5579	1	1 min	16
		Phys1-LKSD-UPW-2	5697	3	1 min	49
		Phys1-LKSD-FL70-1	5425	1	1 min	16
		Phys1-LKSD-FL70-2	5387	0	1 min	0
	Spiked LKSD-1	Phys1-Spiked-LKSD-UPW-1	5602	41	1 min	651
		Phys1-Spiked-LKSD-UPW-2	4269	40	1 min	635
		Phys1-Spiked-LKSD-FL70-1	5331	47	1 min	746
		Phys1-Spiked-LKSD-FL70-2	5329	51	1 min	810
**Physical two-step extraction**	LKSD-1	Phys2-LKSD-UPW-1	5963	1	1 min	16
		Phys2-LKSD-UPW-2	5900	1	1 min	16
		Phys2-LKSD-FL70-1	5786	1	1 min	16
		Phys2-LKSD-FL70-2	5845	2	1 min	32
	Spiked LKSD-1	Phys2-Spiked-LKSD-UPW-1	5923	45	1 min	714

		Phys2-Spiked-LKSD-UPW-2	4303	50	1 min	794
		Phys2-Spiked-LKSD-FL70-1	5776	54	1 min	857
		Phys2-Spiked-LKSD-FL70-2	4423	53	1 min	841
**Colloidal extraction**	LKSD-1	Coll-LKSD-UPW-1	12218	2	2 min	32
		Coll-LKSD-UPW-2	12168	1	2 min	16
		Coll-LKSD-FL70-1	11707	4	2 min	64
		Coll-LKSD-FL70-2	11695	2	2 min	32
	Spiked LKSD-1	Coll-Spiked-LKSD-UPW-1	6213	67	1 min	1064
		Coll-Spiked-LKSD-UPW-2	4412	56	1 min	889
		Coll-Spiked-LKSD-FL70-1	5970	72	1 min	1143
		Coll-Spiked-LKSD-FL70-2	4433	51	1 min	810
**Physical one-step extraction**	LKSD-1	LKSD-FL70-1	29823	42	5 min	133
		LKSD-FL70-2	29512	48	5 min	152
**Pt-Spiking solution**	Before centrifugation	Pt-solution-UPW-1	1597	1328	1 min	21079
		Pt-solution-UPW-2	1554	1329	1 min	21095
	After centrifugation	Pt-solution-cent-UPW-1	225	83	1 min	1317
		Pt-solution-cent-UPW-2	264	72	1 min	1143
**Blanks**	Blank	Blank-UPW	89	0	1 min	–
		Blank-FL70	535	1	1 min	16

ICP-TOF-MS operational parameters

Table S3. ICP-TOF-MS operational parameters on the measurement day of each extraction procedure.

	**Colloidal Extraction**	**Physical One-Step Extraction**	**Physical Two-Step Extraction**
**Plasma power [W]**	1550	1550	1550
**Cooling gas flow [$l \cdot min^{-1}$]**	14	14	14
**Auxiliary gas flow [$l \cdot min^{-1}$]**	0.8	0.8	0.8
**Nebulizer flow [$l \cdot min^{-1}$]**	1.30	1.43	1.43
**Sample flow rate [$\mu l \cdot min^{-1}$]**	300	300	300
**TOF repetition rate [kHz]**	22	22	22
**Detected mass range**	14 – 275 m/z	14 – 275 m/z	14 – 275 m/z
**Masses notched**	$^{23}Na^+$, $^{30}NO^+$, $^{32}O^+$, $^{40}Ar^+$	$^{23}Na^+$, $^{30}NO^+$, $^{32}O^+$, $^{40}Ar^+$	$^{23}Na^+$, $^{30}NO^+$, $^{32}O^+$, $^{40}Ar^+$
**Data acquisition**	Continuous mode	Continuous mode	Continuous mode
**TOF integration time [ms]**	3	3	3
**Transport efficiency [%]**	21.0	20.5	20.5
**Oxides (CeO^+/Ce^+) [%]**	0.21	0.26	0.26
**Doubly charged (Ba^{++}/Ba^+) [%]**	0.90	1.59	1.59

Table S4. Apex Omega desolvation unit operation parameters.

**Argon flow rate [$L \cdot min^{-1}$]**	2.85
**Nitrogen flow rate [$L \cdot min^{-1}$]**	0.0

**Peltier Cooler temperature [ºC]**	3.3
**Spray Chamber temperature [ºC]**	140.0
**Desolvator temperature [ºC]**	155.3
**Peristaltic pump speed [rpm]**	17.0
**Peristaltic pump flow [µL·min⁻¹]**	600.0

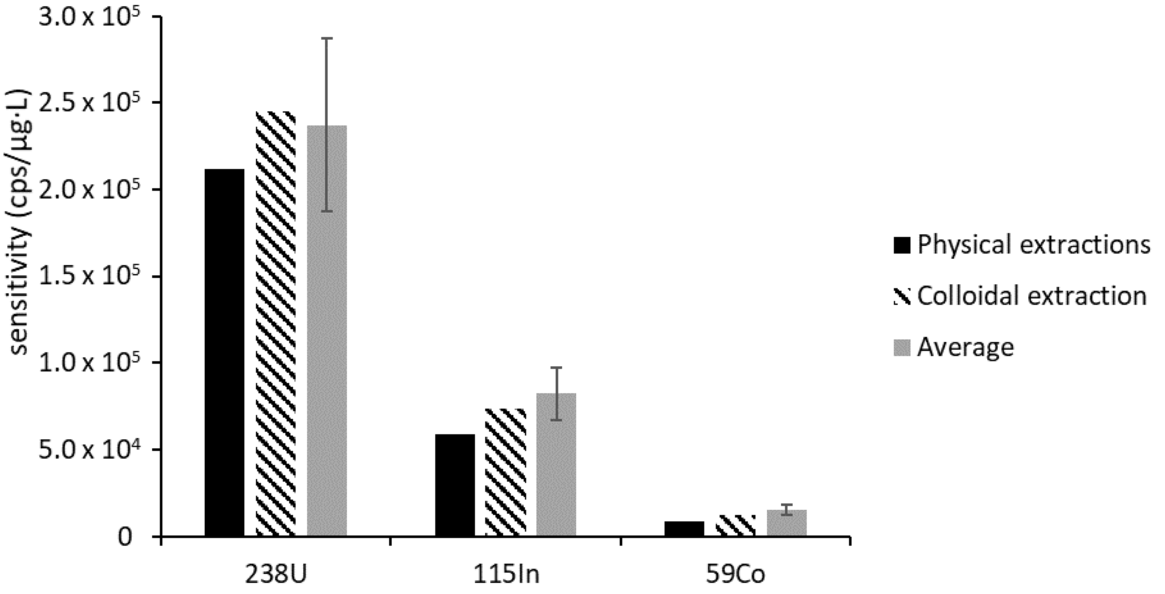

Figure S1. Sensitivity expressed as counts per second (cps/µg·L^{-1}). The black columns represent the sensitivities measured on the physical extractions measurement day, the dashed columns the sensitivities on the colloidal extraction measurement day, and the grey columns the average sensitivities achieved by the ICP-TOF-MS for 6 months.

Table S5. Limits of detection of the monitored analytes converted from concentration $LOD_{concentration}$ (ng.L^{-1}) into mass LOD_{mass} (fg) and particle diameter $LOD_{diameter}$ (nm) according to Pace et al. (2011) and Lee et al. (2014). $LOD_{diameter}$ was determined assuming a pure particle and spherical shape.

Analyte	Isotope	$LOD_{concentration}$ (ng.L^{-1})	LOD_{mass} (fg)	$LOD_{diameter}$ (nm)
**Na**	23	271	0.834	118
**Mg**	24	80.3	0.250	64.7
**Al**	27	181	0.557	73.1
**Si**	28	74700	229	573
**Ti**	48	51.4	0.158	40.6
**V**	51	19.1	0.0588	27.3
**Cr**	52	55.9	0.172	35.8
**Mn**	55	15.7	0.0484	23.2
**Fe**	56	367	1.13	64.9
**Ni**	58	24.9	0.0764	25.4
**Co**	59	11.8	0.0364	19.9
**Cu**	63	35.6	0.109	28.6
**Zn**	64	18.4	0.0565	24.7
**As**	75	20.7	0.0635	27.7
**Y**	89	1.06	0.00327	11.2

**Zr**	90	5.59	0.0172	17.1
**Mo**	98	6.56	0.0202	15.6
**Ru**	102	9.51	0.0293	16.5
**Rh**	103	2.81	0.00865	11.0
**Pd**	106	9.61	0.0296	16.7
**Ag**	107	6.55	0.0201	15.4
**Cd**	114	4.88	0.0150	14.9
**Sn**	120	7.29	0.0224	18.1
**Sb**	121	14.6	0.0450	23.4
**Te**	130	82.2	0.253	42.6
**Ba**	138	15.1	0.0465	29.1
**La**	139	0.70	0.00217	8.77
**Ce**	140	1.95	0.00600	11.9
**Pr**	141	0.47	0.00145	7.43
**Ir**	193	2.02	0.00622	8.06
**Pt**	195	4.55	0.0140	10.8
**Au**	197	9.27	0.0385	14.1
**Pb**	208	3.09	0.00950	11.7
**Bi**	209	8.20	0.0252	17.0

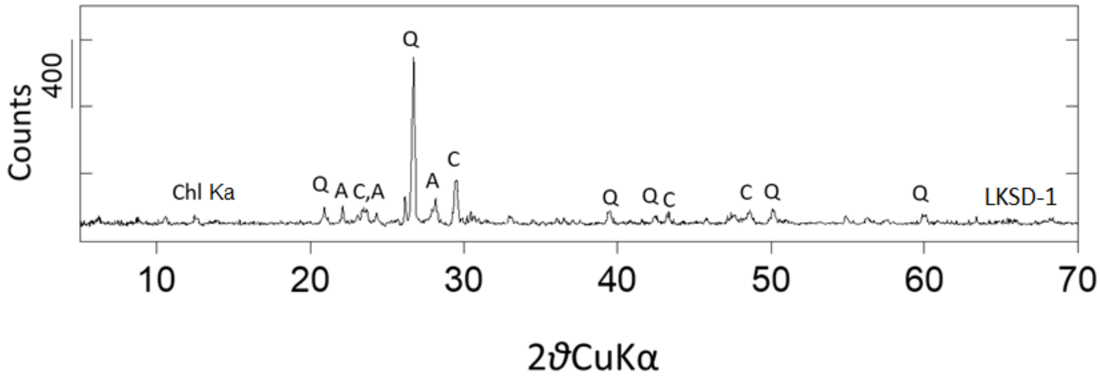

Figure S2. X-ray diffraction pattern of the sediment LKSD-1 sample. Chl: Chlorite; Ka: Kaolonite; Q: Quartz; A: Albite; C: Calcite.

Table S6. Total organic carbon (TOC), inorganic carbon (TIC), and total carbon (TC) content in the sediment sample.

	**TOC (%)**	**TIC (%)**	**TC (%)**
**LKSD-1**	10.7	2.6	13.3

Platinum mass distribution in the Pt-containing NPs

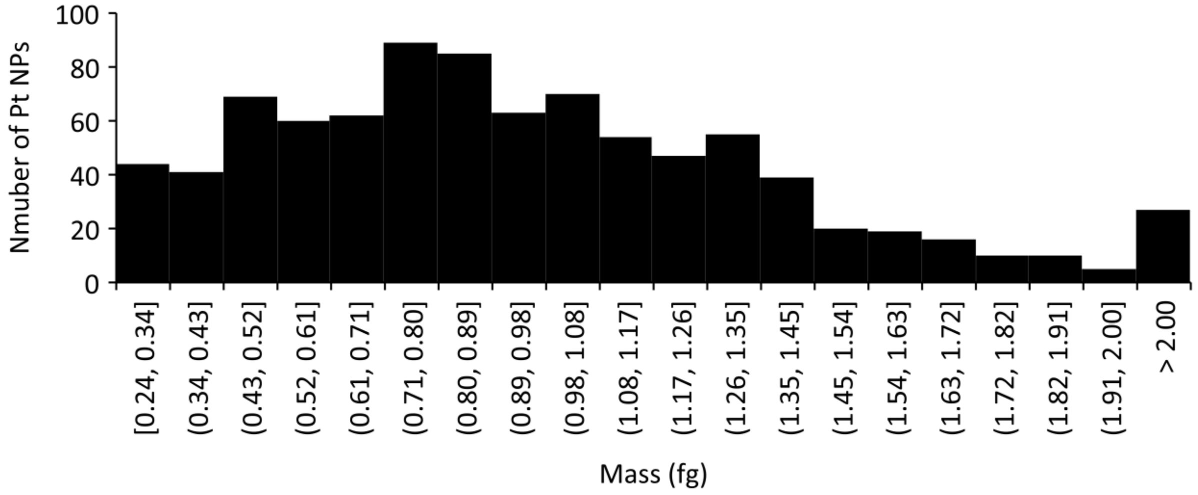

Figure S3. Pt mass distribution of Pt NPs in the undiluted spiking solution.

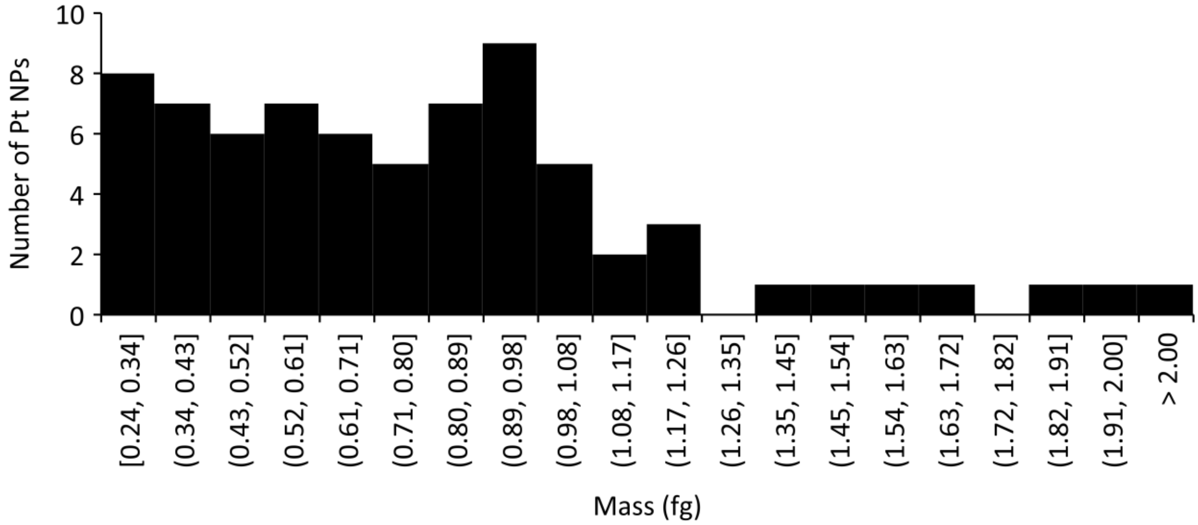

Figure S4. Pt mass distribution of Pt NPs in the spiking solution after centrifugation to a 1.5 µm cut-off, resuspension, and dilution by a factor of 200 in UPW.

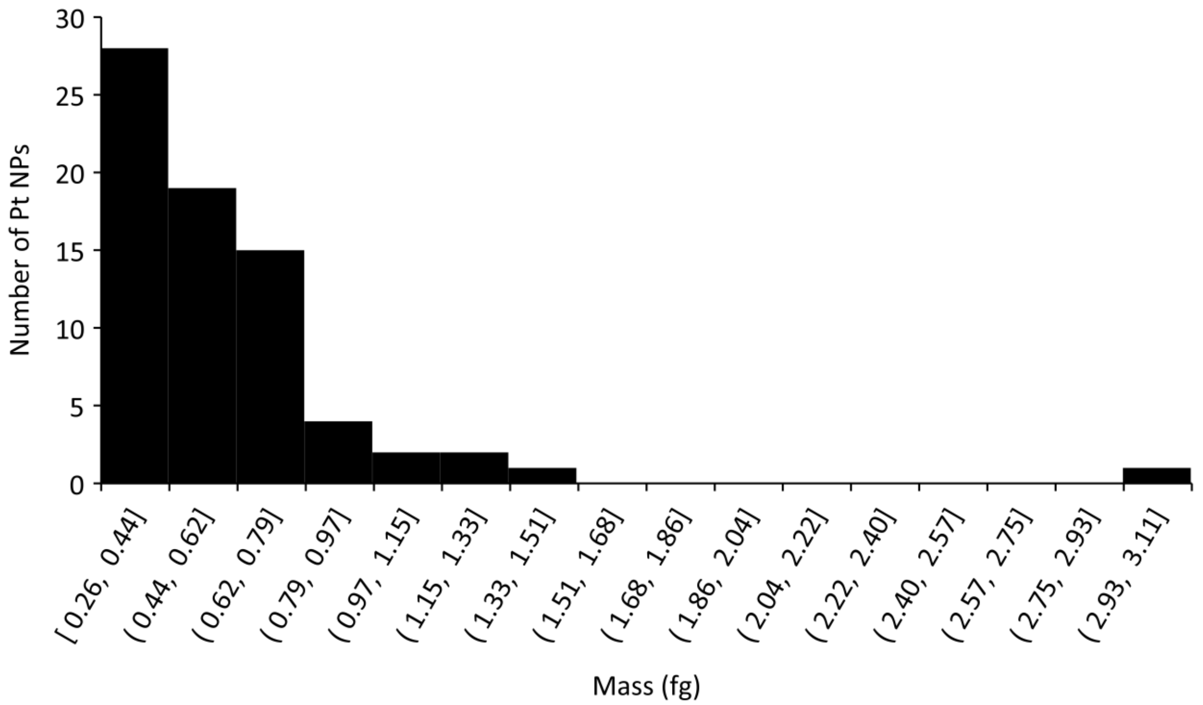

Figure S5. Pt mass distribution in the Pt NPs of the Pt-spiked sediment sample dispersion. The NPs were extracted by colloidal extraction, dispersed in FL70, and acquired over 1 minute.

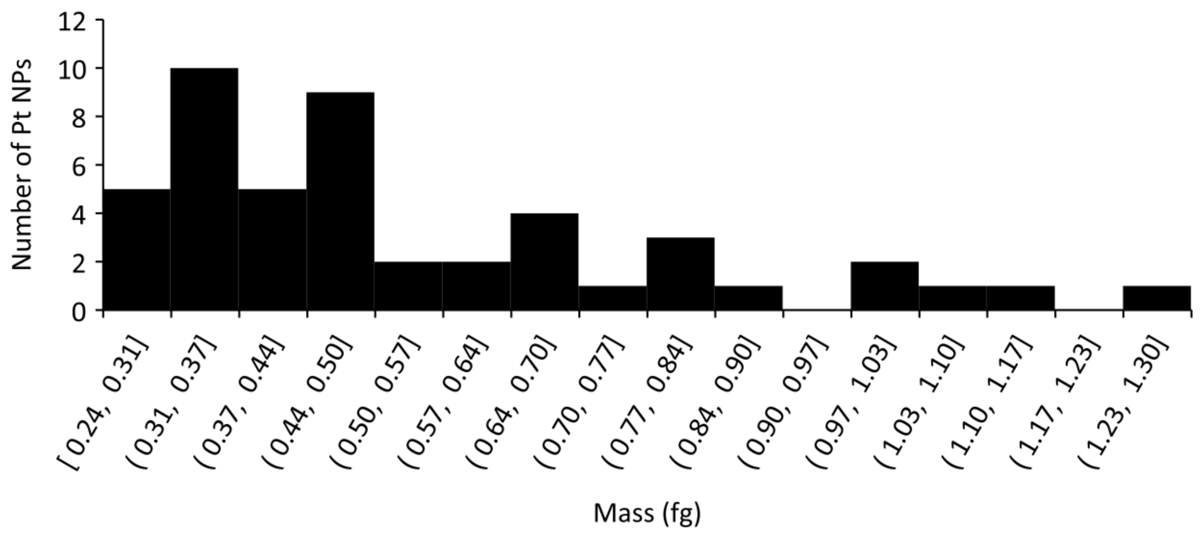

Figure S6. Pt mass distribution in the Pt NPs of the Pt-spiked sediment sample dispersion. The NPs were extracted by physical one-step extraction, dispersed in FL70, and acquired over 1 minute.

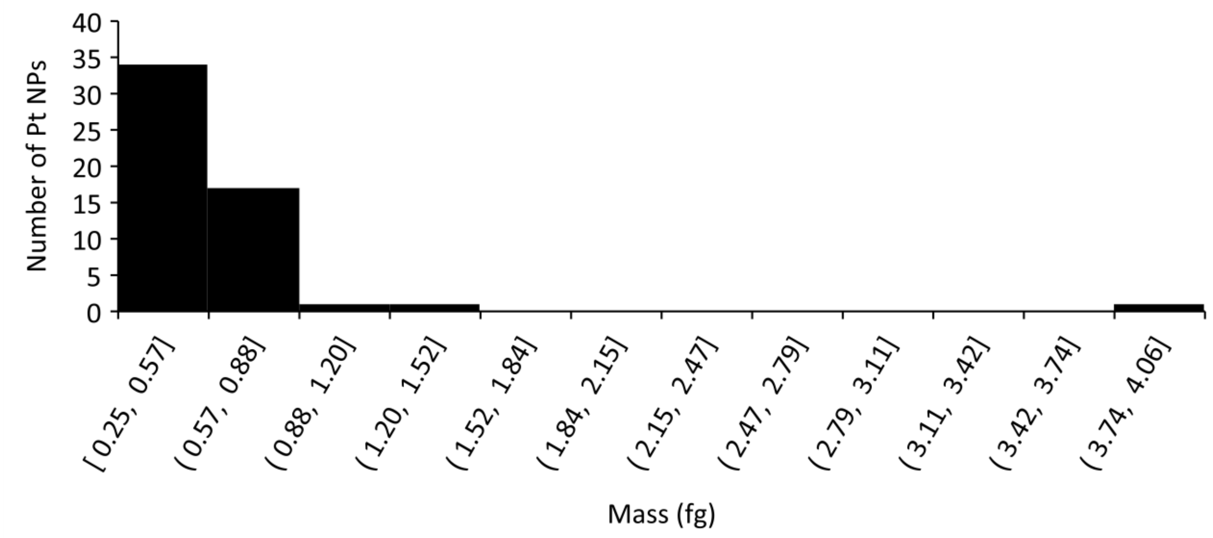

Figure S7. Pt mass distribution in the Pt NPs of the Pt-spiked sediment sample dispersion. The NPs were extracted by physical two-step extraction, dispersed in FL70, and acquired over 1 min.

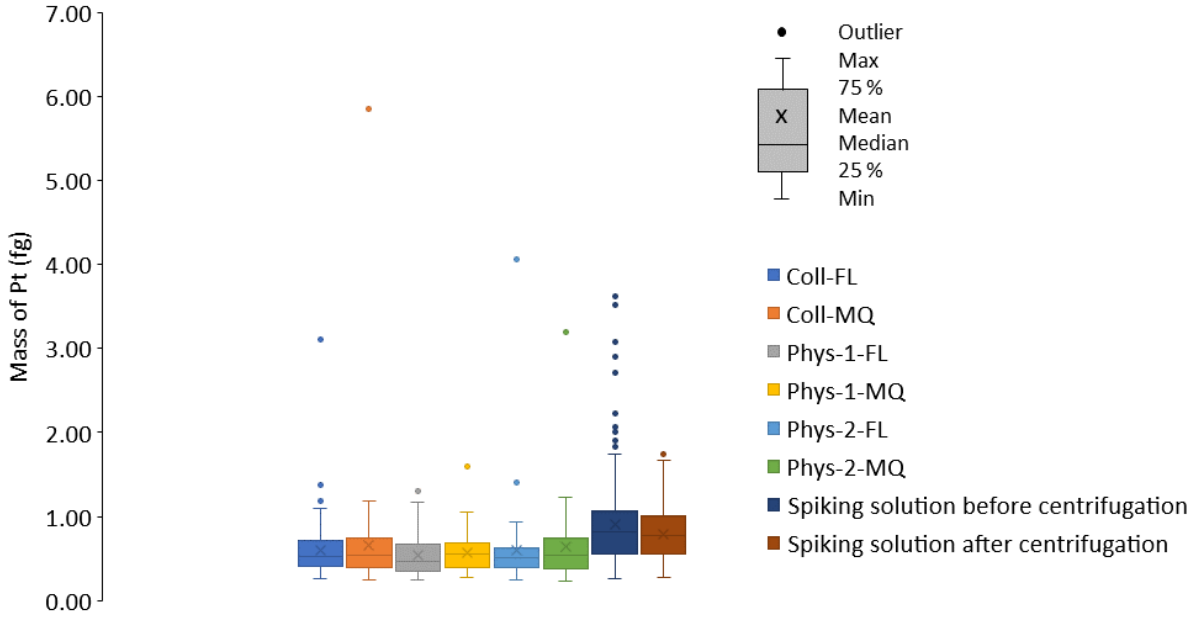

Figure S8. Summary of the mass distribution of Pt in the Pt NPs of the Pt-spiked sediment samples of each extraction procedure and the Pt NPs spiking solution before and after centrifugation.

Table S7. Dissolved ions concentration represented as counts per second (cps) of selected major elements in the pristine sediment sample following particle extraction for each extraction procedure. The samples were dispersed in FL70.

	Aluminum	Calcium	Iron	Magnesium
**Physical one-step extraction**	7.53×10^4	2.25×10^5	1.67×10^5	1.73×10^5
**Physical two-step extraction**	9.88×10^4	1.95×10^5	1.96×10^5	7.47×10^4
**Colloidal extraction**	1.12×10^5	1.47×10^5	3.14×10^5	1.35×10^5

Elemental composition of Pt-containing nanoparticles in the Pt-spiked sediment samples

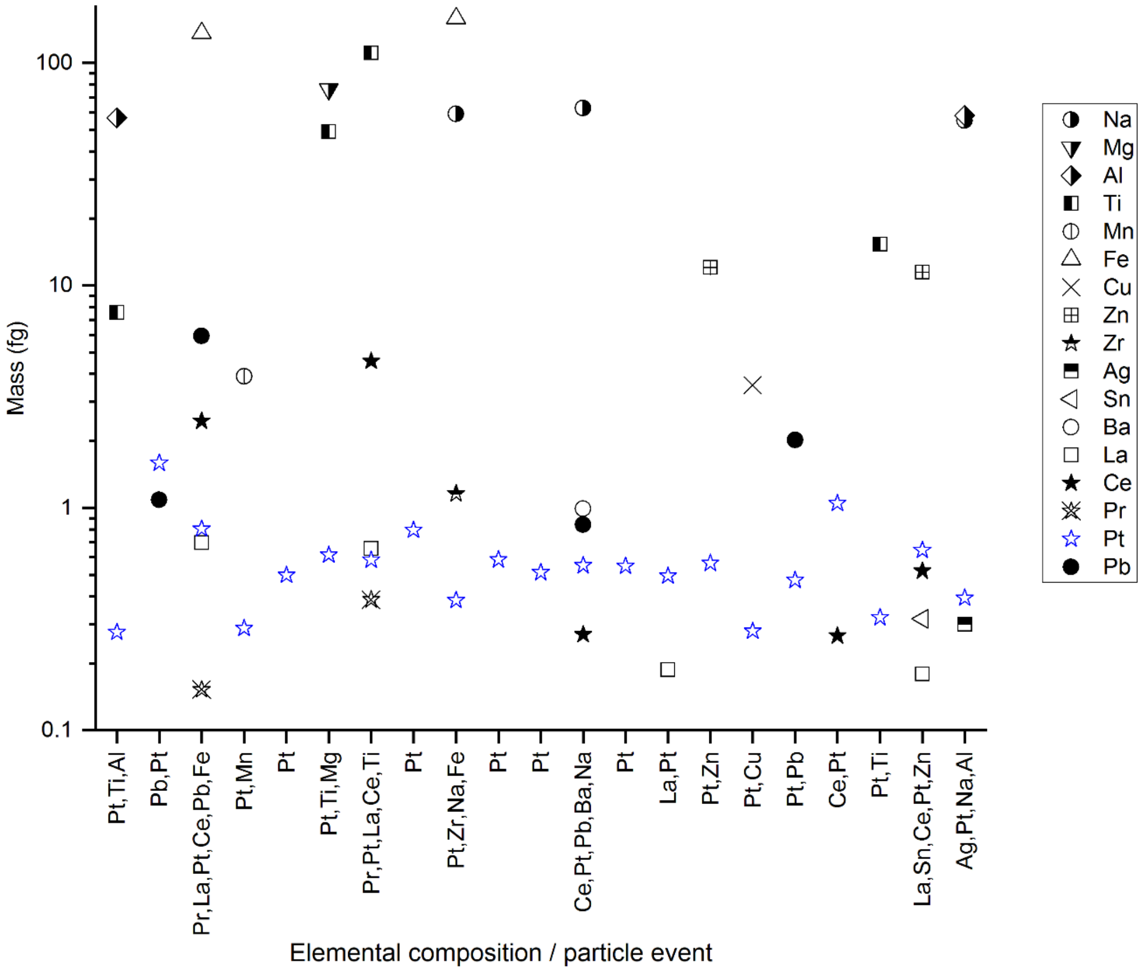

Figure S9. Pt-containing NP elemental compositions in the Pt-spiked sediment sample dispersion. The NPs were extracted by physical one-step extraction, dispersed in UPW, and acquired over 30 seconds out of 1 minute total acquisition time. The *y*-axis indicates the mass of each element and the *x*-axis the elemental composition.

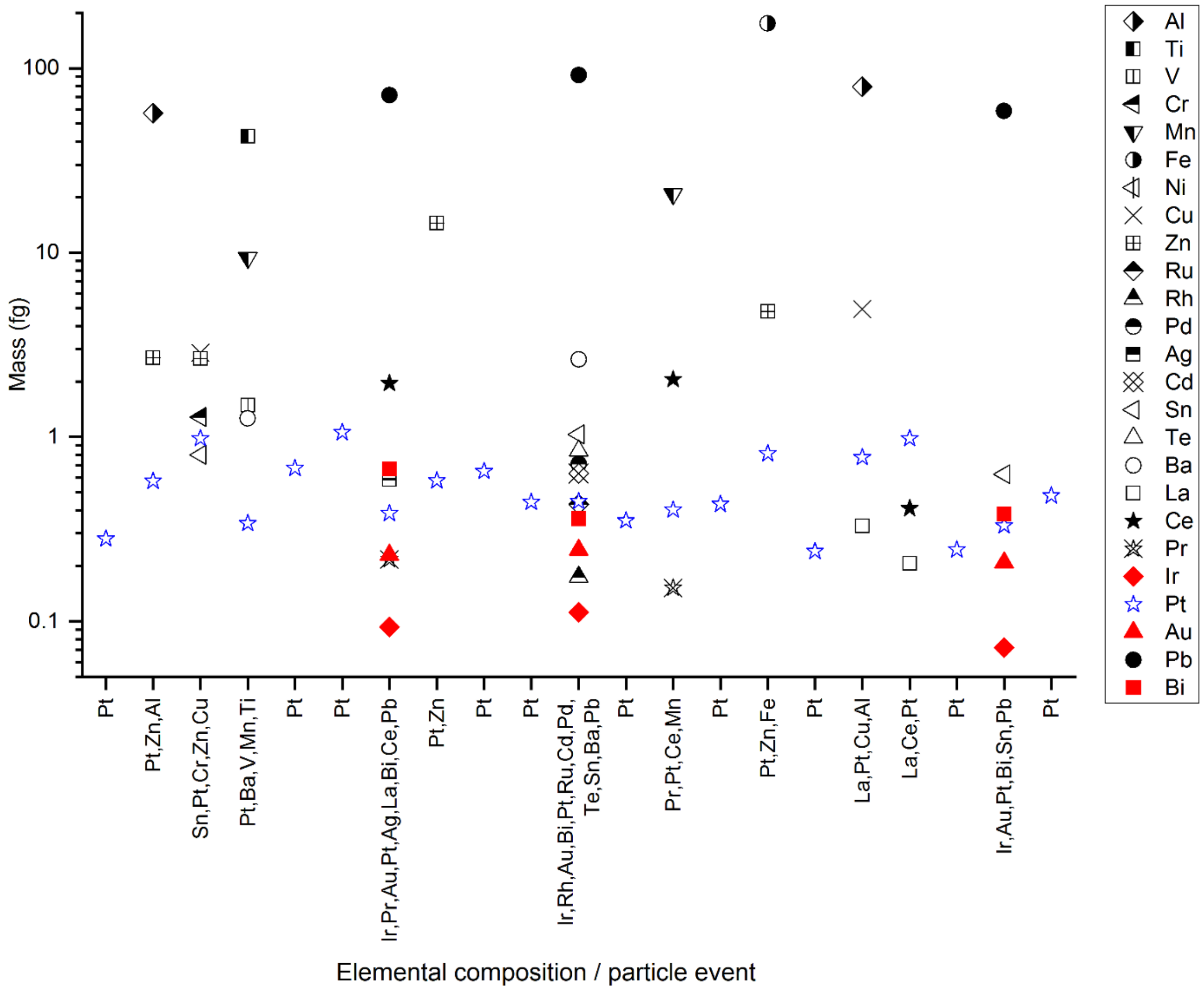

Figure S10. Pt-containing NP elemental compositions in the Pt-spiked sediment sample dispersion. The NPs were extracted by physical one-step extraction, dispersed in FL70, and acquired over 30 seconds out of 1 minute total acquisition time. The *y*-axis indicates the mass of each element and the *x*-axis the elemental composition.

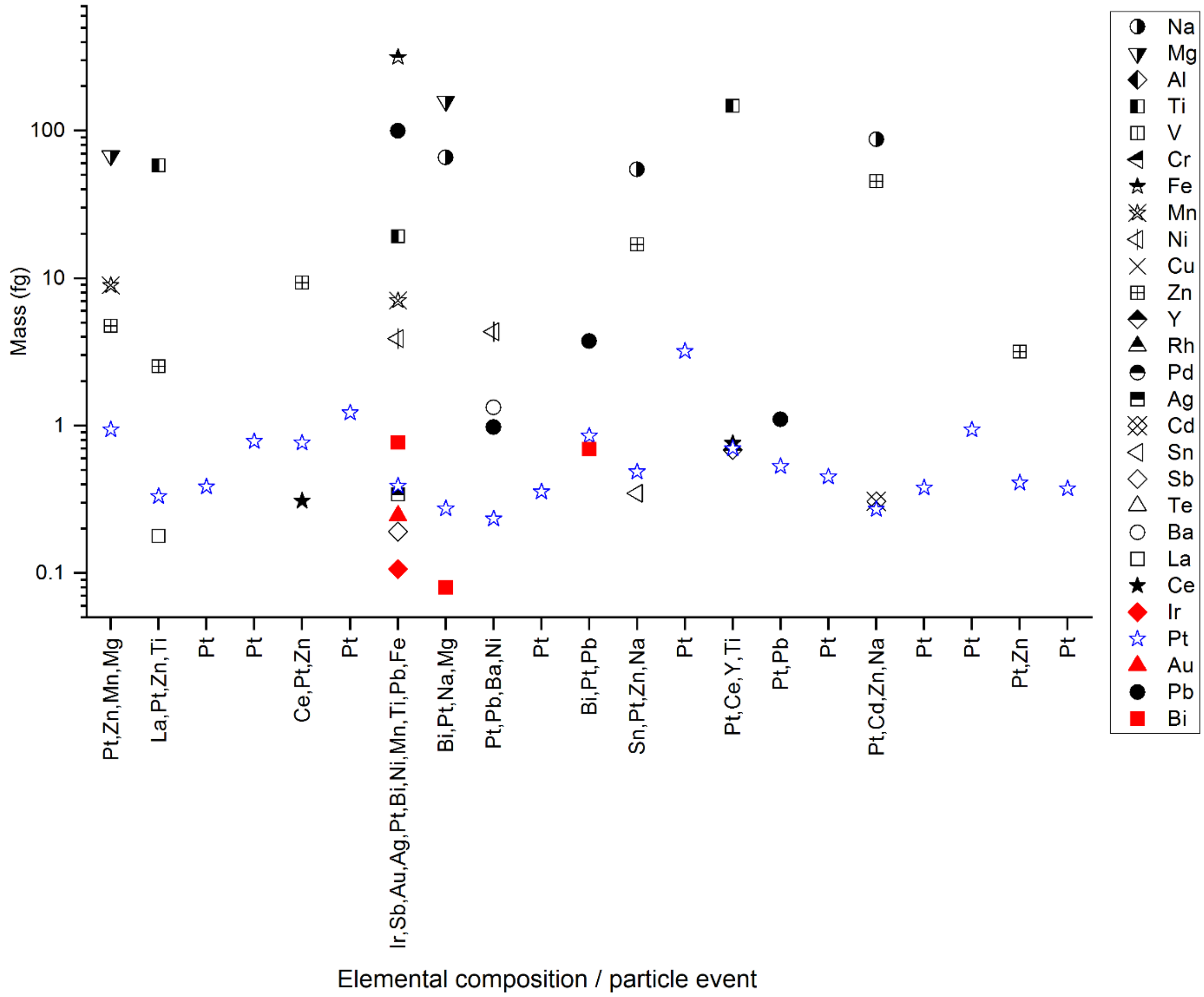

Figure S11. Pt-containing NP elemental compositions in the Pt-spiked sediment sample dispersion. The NPs were extracted by physical two-step extraction, dispersed in UPW, and acquired over 30 seconds out of 1 minute total acquisition time. The *y*-axis indicates the mass of each element and the *x*-axis the elemental composition.

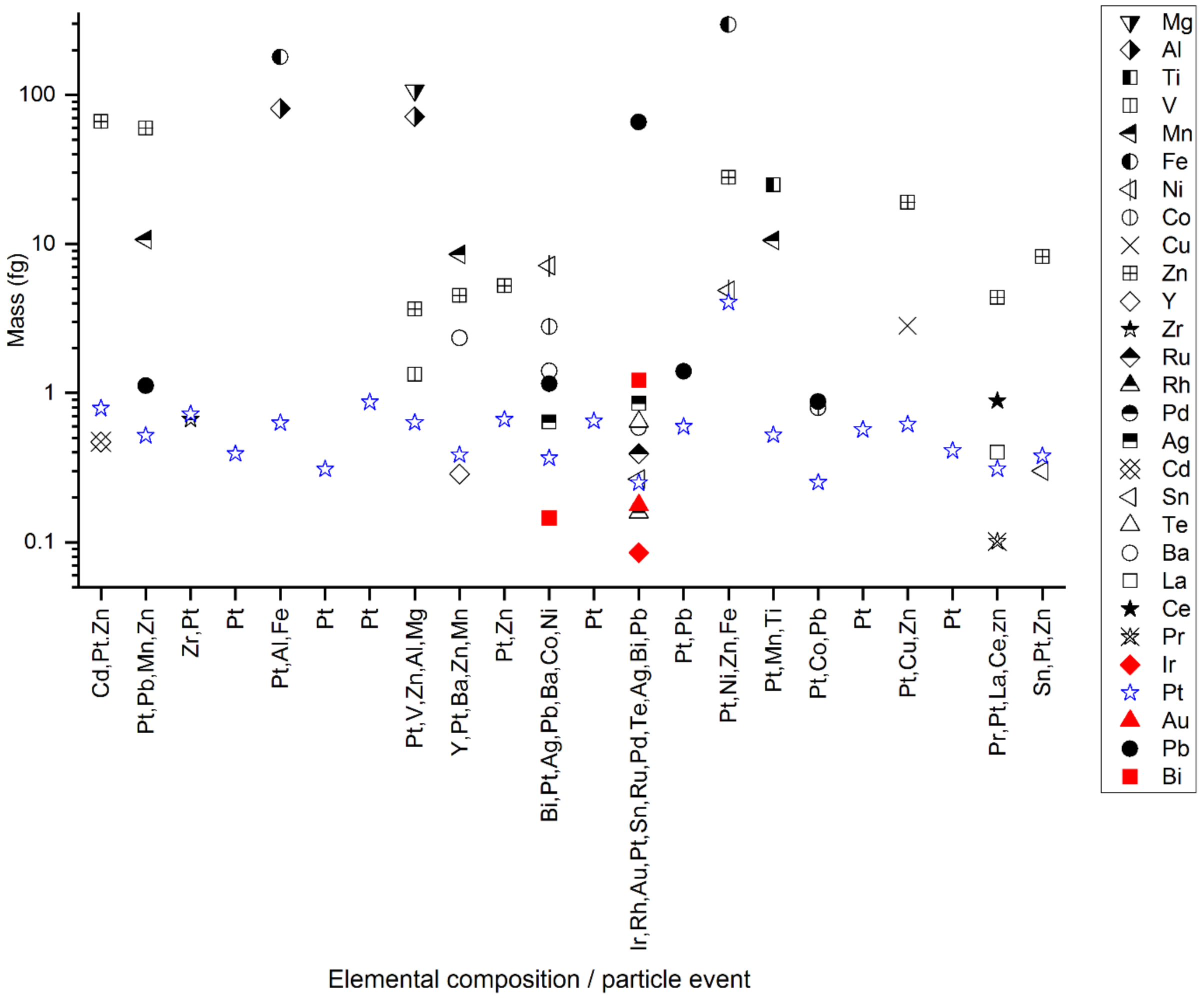

Figure S12. Pt-containing NP elemental compositions in the Pt-spiked sediment sample dispersion. The NPs were extracted by physical two-step extraction, dispersed in FL70, and acquired over 30 seconds out of 1 minute total acquisition time. The y-axis indicates the mass of each element and the x-axis the elemental composition.

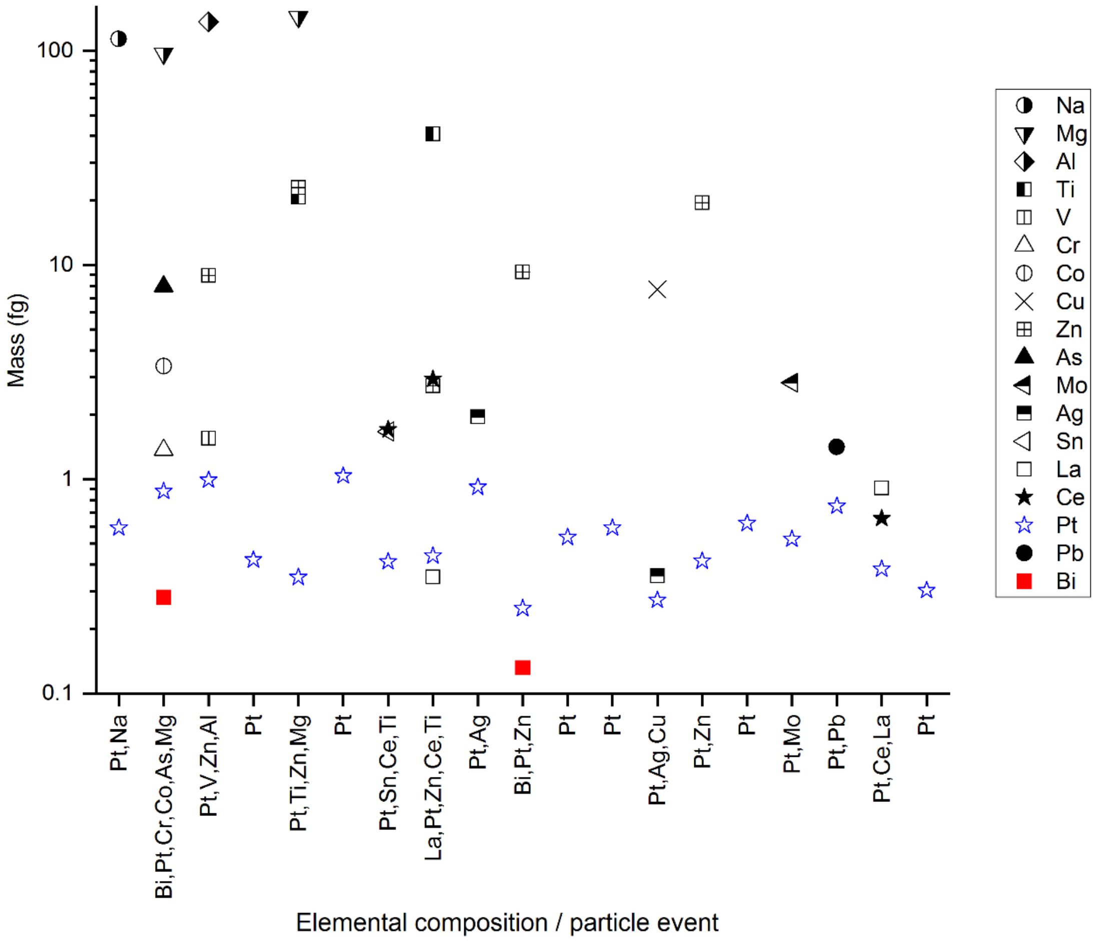

Figure S13. Pt-containing NP elemental compositions in the Pt-spiked sediment sample dispersion. The NPs were extracted by colloidal extraction, dispersed in UPW, and acquired over 30 seconds out of 1 minute total acquisition time. The *y*-axis indicates the mass of each element and the *x*-axis the elemental composition.

Temporal investigation of Pt nanoparticle recoveries

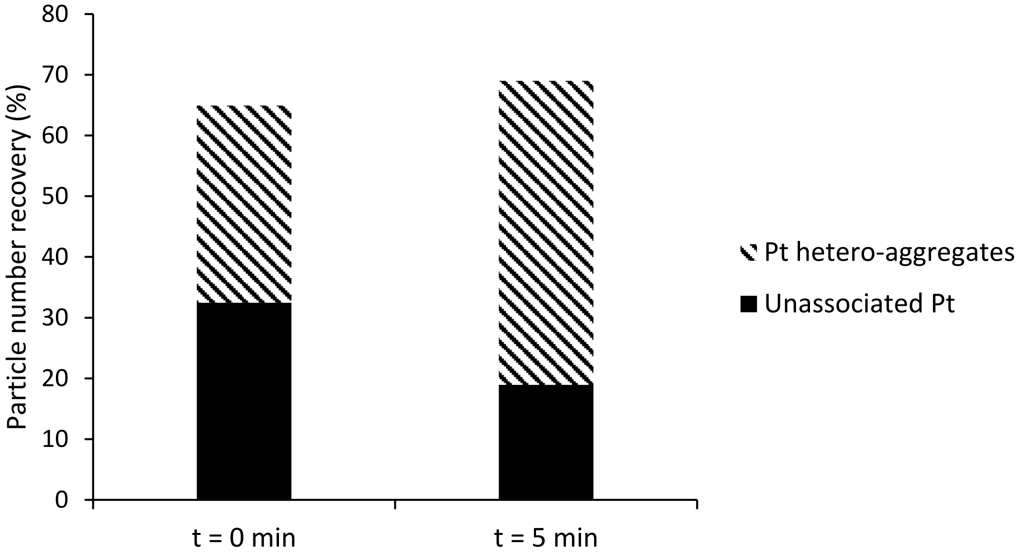

Figure S14. Particle number recoveries (#%) of spiked Pt NPs from sediment samples, as single un-associated Pt NPs (black columns) and as Pt NPs hetero-aggregated with other sediment particles (dashed columns), at t = 0 min and t = 5 min after sample redispersion (shaking and sonication). The NPs were extracted by physical one-step extraction and dispersed in FL70 prior to ICP-TOF-MS analysis.
